# Quantitative Assessment of the Entry through Mechanical Transport in Aircraft of Rift Valley Fever Virus-Infected Mosquitoes into Previously Unaffected Areas

**DOI:** 10.3390/pathogens10050541

**Published:** 2021-04-30

**Authors:** Maria-Eleni Filippitzi, Claude Saegerman

**Affiliations:** 1 Veterinary Epidemiology Unit, Department of Epidemiology and Public Health, Sciensano, 1050 Brussels, Belgium; 2 Research Unit of Epidemiology and Risk Analysis Applied to Veterinary Sciences (UREAR-ULiège), Fundamental and Applied Research for Animals and Health (FARAH) Center, Faculty of Veterinary Medicine, University of Liege, 4000 Liège, Belgium; claude.saegerman@ulg.ac.be

**Keywords:** Rift Valley Fever, arbovirus(es), mosquito(es), risk analysis, transport

## Abstract

(1): Rift Valley Fever (RVF) is a zoonotic disease of significant international health concern and considered as an emerging risk to Europe, where no RVF outbreaks in humans or animals have been reported so far. (2): Using a stochastic approach, we estimated the risk of RVF virus (RVFV) introduction during the period of May to October (the period when mosquito populations, including RVFV potential vectors, are present in European countries), into previously unaffected areas (e.g., United Kingdom, UK) via virus-carrying vectors traveling in commercial aircraft from RVF-affected countries (e.g., East Africa); (3): On average *N* = 68 (95% CI: 0–337), RVF-virus-infected mosquitoes are estimated to be mechanically transported by planes (with *N* = 0 as most likely), in direct flights from RVF-affected East African countries to the UK, between May and October. This estimate is considered as low but not negligible. The model developed should be easily scaled up to other European countries by amending appropriately country-specific variables (e.g., number of flights between countries) in order to map the areas/airports of higher risk and inform risk management per country accordingly and to adopt risk-mitigation measures.

## 1. Introduction

Rift Valley Fever (RVF) is a disease of significant international health concern and an emerging risk of interest to Europe, where no RVF outbreaks in humans or animals have been reported so far. This infectious zoonotic disease is caused by Rift Valley Fever virus (RVFV), belonging to the Phlebovirus genus (Bunyaviridae), a group of ribonucleic acid (RNA) viruses. The virus affects mainly sheep, goats, and cattle; causing abortions and a high neonatal mortality rate. The main transmission mechanism of RVFV in ruminants is a bite from infected mosquito vectors, while the virus has the potential to also be spread by other arthropods such as sand flies [1]. Susceptibility varies considerably among ruminants of different ages and breeds, with younger animals being more susceptible [2,3]. Humans become infected through direct contact with blood, body fluids, or aborted materials from infected animals, or through mosquito bites [4,5]. There is some evidence that humans can become infected with RVF by ingesting unpasteurized milk or uncooked meat from infected animals. The majority of human infections lead to a mild flu-like syndrome, but a small percentage (7–8%) leads to more severe manifestations, including fatal hemorrhagic disease [4,6,7]. Outbreaks of RVF can result in significant economic losses [8]. Indicatively, the outbreaks that occurred in Kenya in 2007 have been estimated to have caused over US $32 million in damage to the Kenyan economy through severe losses to agriculture, human health, and other sectors, such as transport [9].

RVFV, which was first recognized in Kenya in 1931, has been found today in countries across Africa, the Arabian Peninsula, and islands in the Indian Ocean, including Madagascar, Comores, and Mayotte [10,11]. The virus has a broad host range and can be transmitted by at least 30 different mosquito species—some of which are found in Europe, Australasia, and the Americas [2,12,13]. Given the increasing movements of people, animals, plants, and goods worldwide, there is a risk of RVFV and its vectors spreading from their current range into regions of the world where they have not been previously reported. Previous qualitative risk assessments have assessed the following potential pathways of introduction of RVF-virus in the United Kingdom (UK) (APHA, unpublished), the Netherlands (Hoek, M., Central Veterinary Institute, The Netherlands, personal communication), the European Union (EU) [2], and the United States (US) [14,15]: competent animal hosts, animal products, pests, RVF vectors, human hosts, vaccines, and escape from laboratories. According to these previous qualitative and generic quantitative risk assessments for RVFV introduction to a country [11], the pathways of non-negligible risk are the mechanical transport of virus-carrying vectors confined within aircrafts and ship cargo holds, and the legal or illegal importation of viremic hosts and animal products from RVF-affected countries.

The strength and added value of quantitative approaches, when it is possible to perform them in addition to qualitative ones, lie in their usefulness for an informed and precise illustration of a risk for policy, and also for highlighting the lack of data (including quality data) in areas that require further research (including surveillance). Therefore, we were interested in taking the previous research a step further and quantitatively assessing, using a stochastic approach, the risk of RVFV introduction via the pathways previously assessed as non-negligible. Due to the lack of quantitative data considered as pivotal to build a risk model as realistic as possible for the non-negligible pathways, this study aimed at quantitatively assessing the risk of RVFV introduction into previously unaffected areas via virus-carrying vectors traveling in commercial aircraft from RVF-affected countries. Using the United Kingdom as an example of an RVF-free European country receiving flights from affected countries, considering African countries as examples of RVF-affected countries, and focusing on mosquitoes, the study attempted to build an approach applicable to a broader extent of cases (i.e., other European countries).

## 2. Results

Table 1 presents the estimated results of the model regarding its output; that is, the number of RVFV-infected mosquitoes mechanically transported by planes, in direct flights from RVF-affected East African countries to the UK, during the period of May to October, and given that an epidemic occurs (*N*). Table 1 further presents the estimates of the model over the input parameters: the probability of a random mosquito being infected with RVFV in an RVF-affected East African country, given that an epidemic occurs (*p*), the number of mosquitoes mechanically transported in a direct flight from an RVF-affected East African country to the UK (*N*_1_), and the number of mosquitoes mechanically transported by planes, in direct flights from RVF-affected East African countries to the UK (*N_T_*) for the period of May to October.

**Figure 1:**
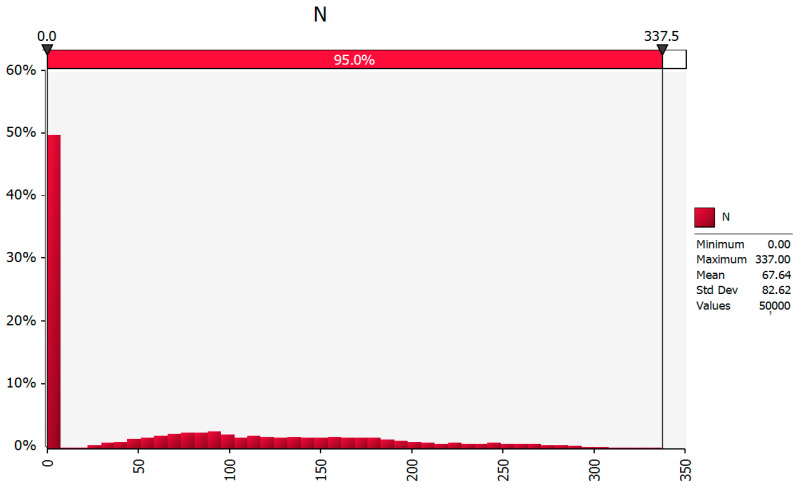
Distribution (*x* axis) and relative frequency (*y* axis, %) of the number of RVFV-infected mosquitoes mechanically transported by planes, between May and October, in direct flights from RVF-affected countries to European countries (*N*). For illustration purposes, the example of flights from East African affected countries to the UK were considered.

According to this model, the mosquitoes, when present in such direct flights, would be expected to be present in small numbers aboard an aircraft, with a mode (most likely) value of *N*_1_ = 0, and a mean value of *N*_1_ = 1.1 (95% CI: 0–3). Moreover, this model estimated that, given our assumptions, when an outbreak occurs, the total number of RVFV-infected mosquitoes that are mechanically transported by planes, in direct flights from RVF-affected East African countries to the UK, between May and October, has a mode (most likely) value of *N* = 0 (i.e., no infected mosquitoes are found in these flights) and a mean (average) value of *N* = 68 (95% CI: 0–337). Figure 1 presents visually the distribution and the relative frequency representing *N* for this model. In detail, according to this model, the most frequent event estimated to occur (with almost 50% estimated relative frequency) is that no infected mosquitoes are transferred aboard aircraft from and to the countries in focus. However, the model also shows that, albeit with much lower estimated frequency (<10%), higher total numbers of infected mosquitoes (with *N*_max_ = 337) could be transferred via this route.

**Figure 2:**
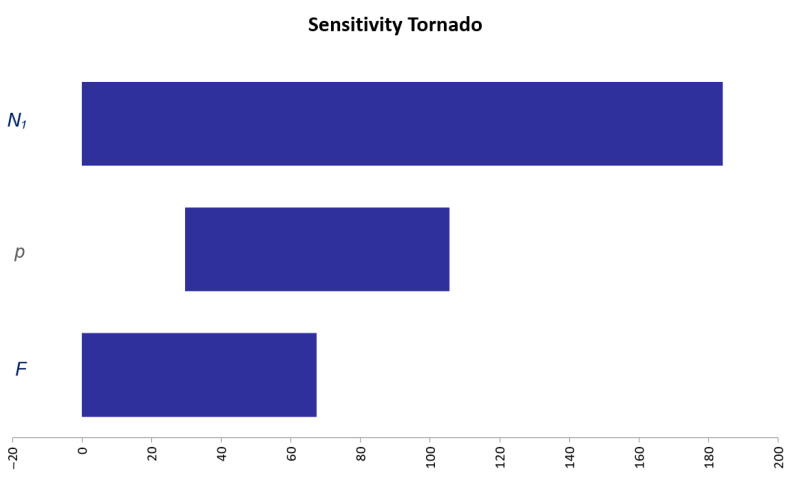
Results of sensitivity analysis of the model output, i.e., the number of RVFV-infected mosquitoes mechanically transported by planes during the period of May to October, in direct flights from RVF-affected countries to an EU country (*N*). Legend: *N_1_* is the number of mosquitoes mechanically transported in a direct flight from an RVF-affected country (in this example from East Africa) to a European country (in this example to the UK). *p* is the probability of a random mosquito being infected with RVFV in an RVF-affected country. *F* is the average number of direct flights to a European country from RVF-affected countries, during a time period of interest. The *x*-axis of the tornado chart represents the values of the *N* for different values of the independent variables *N*_1_, *p*, *F* (*y*-axis). Each bar represents the range of *N* values produced when each of *N*_1_, *p*, *F* is set to lower bound and upper bound (with the other variables being held constant). The variable that produces the largest range of the *N* values between its lower and upper bound is at the top of the chart.

A sensitivity analysis was also performed. Figure 2 displays the results of the sensitivity analysis graphically represented in the form of a tornado chart. This chart shows the degree to which the output of this model (i.e., the number of infected mosquitoes mechanically transported in the flights of interest) is sensitive to the specified independent variables considered (i.e., the number of mosquitoes mechanically transported in a direct flight (*N_1_*), the probability of a random mosquito being infected with RVFV (*p*) and the number of direct flights to a European country (e.g., the UK) from RVF-affected countries (e.g., of East Africa) (*F*). As shown in Figure 2, the sensitivity analysis revealed that the output of this model (*N*) is most sensitive to *N_1_*, followed by *p* and *F*.

## 3. Discussion

This model estimated a low, but not negligible, number of RVFV-infected mosquitoes being mechanically transported by planes, in direct flights from RVF-affected East African countries to the UK (*N*). Moreover, the sensitivity analysis showed that the number of mosquitoes mechanically transported in a random direct flight (*N*_1_) influences the output of this model (*N*) the most. Taking into consideration the underestimation of the total number of flights from RVF-affected countries arriving to the UK (e.g., the UK also receives on average 1915 flights from West Africa from May to October), it could be expected that the total risk of RVF introduction to the UK through mosquitoes aboard flights is underestimated. Based on these results, it becomes obvious that the implementation of efficient (and passenger-safe) disinsection policies from the airlines, as well as mosquito control at the airports and the surrounding areas (described elsewhere, e.g., [16]) are of utmost importance to minimize the risk of introduction of RVF, but also of other vector-borne diseases. Moreover, given the increased need for quantitative approaches in risk assessment and risk modeling for informing policy and in order to reduce uncertainty of their estimates, there is a need for detailed quantitative data from entomological surveillance inside aircraft, inside and outside airport areas.

During the study, it also appeared that few data were available, especially regarding the prevalence of RVF from entomological studies (*p*). Therefore, the results of available studies with data from affected areas in Sudan and Kenya were used and it was assumed that these results were representative of RVF prevalence in mosquitoes in East Africa. Respective data from studies in western or southern Africa were not identified, a fact that did not allow us to consider distinct scenarios representing different parts of Africa with different epidemiological cycles of RVFV (see spatio-temporal characteristics of RVFV persistence). Therefore, there is an identified need for entomological surveillance data to allow a more precise estimation of prevalence. Together with this, databases and maps should be produced, continuously verified, improved in spatial resolution and regularly updated in order to provide data at the sub-national level as well. However, this model is built in a step-by-step way, offering the possibility of easy amendments and updates upon availability of more data (e.g., from entomological studies).

Another less-influential assumption concerns the use of an approximate number of direct flights from East Africa to the UK (*F*), due to the availability of data of number passengers on these flights. For the estimation of the approximate average number of flights, data before 2020 were used to avoid an underestimation due to the COVID-19 pandemic. Regarding the studies used for the estimation of the number of mosquitoes mechanically transported in a direct flight (*N*_1_), these identified small numbers of mosquitoes and this probably shows a good level of disinsection policy (e.g., for the UK and France). However, since for one study (i.e., for The Netherlands) it was not known whether such policy was in place, different scenarios (disinsection versus non-disinsection) could not be considered at this stage. It should also be mentioned that this model focused on the introduction of infected mosquitoes, without investigating the concept of infectiousness [17], which would concern a following exposure assessment. Nevertheless, according to a generic exposure model developed by EFSA (2020) [11] there is a very high probability (combining host density, vector presence, and proportion of days above temperature threshold of 9.6 °C) for a transmission step following a vector introduction to occur in some European countries, including the UK.

This is the first step-by-step risk model that attempts to assess the risk of RVF introduction to a European country, using a stochastic approach, which estimates the number of infected mosquitoes aboard direct flights. The assessment of the risk of RVF introduction based on the results of this model aligns with the results of qualitative assessments previously published [15]. Furthermore, this model complements nicely with other previously developed generic risk models (i.e., MINTRISK [11,18]. It is also relevant to the recently published assessment of effectiveness of surveillance and control measures in the EU by the European Food Safety Authority [19]. Another strength of this model is that the way it is built permits us to use it also in the case of other countries (e.g., of southern Europe) only by amending appropriately country-specific variables (e.g., number of flights between countries). As a next step, it would be useful to continue this research by allocating the arriving mosquitoes to specific airports and, by using spatial mapping, to identify areas/airports of higher risk and inform risk management per country accordingly. Specifically for the UK, the information provided by such a mapping study together the results of this present work, as well as the recent work by Simons et al. (2019) [20], would be useful and directly applicable in a future overall assessment of this risk for this country. In detail, Simons et al. (2019) [20] have recently developed a modeling methodology to estimate the habitat suitability for presence of mosquito species in the UK deemed competent for RVF. At the same time, it is important to follow a One-Health approach involving also the environment, and couple this information with remote-sensing climate data and with the results from models investigating environmental drivers of RVF emergence in Africa.

## 4. Materials and Methods

### 4.1. Model Framework

There are three components of risk assessment under traditional OIE guidelines [21], namely entry assessment, exposure assessment, and consequence assessment. The model presented here only focuses on an entry assessment (i.e., until the point at which the virus is introduced into a country via the pathway of infected mosquitoes aboard aircraft). It does not consider the potential following exposure of humans, livestock, or wildlife to the virus and the subsequent consequences given incursion.

### 4.2. Considerations and Assumptions

A number of considerations and assumptions were initially taken into account, in order to build our model in the most representative way. These considerations concern the countries affected by the disease, the vectors of RVFV, the spatio-temporal characteristics of RVFV in the affected areas, and the flights originating from affected countries. Their detailed description follows.

#### 4.2.1. Affected Countries

The virus is endemic in tropical regions of eastern, western, and southern Africa. A list of countries where the most notable outbreaks of RVF have been recorded in Africa and in the Arabian Peninsula has been presented elsewhere, e.g., [11]. The most recent outbreaks that also involved multiple human cases occurred in Mayotte in 2018–2019 [22].

The study specifically focuses on African countries with currently or previously reported endemic disease and substantial outbreaks, or that are known to have some cases (including human ones), periodic isolation of the virus, or serologic evidence of RVFV. For the purposes of this study, we chose to take into consideration these countries as a whole, and not just the regions or districts where cases or outbreaks have occurred. The logic behind this assumption is that, even if cases are reported from certain regions of a country, it cannot be excluded that the virus can reach the area close to the airport(s) of the country. This could occur either via virus-carrying vectors (mechanical transport or movement within the limits of their flight capacity [23]) and/or via viremic hosts transported due to legal or illegal trade purposes or migration [24]. These hosts could be bitten by competent (i.e., vector with biological suitability to transmit the pathogen) and/or abundant disease vectors present at the area close to the airport(s), which could become infected. Additionally, from the point of view of data availability, a number of outbreaks have only been reported at national level and could not be linked to a specific district [3,25,26].

#### 4.2.2. Vectors

RVFV has been identified so far in over 30 species of mosquitoes from seven different genera (*Aedes*, *Anopheles*, *Coquillettidia*, *Culex*, *Eretmapoites*, *Mansonia*, and *Ochlerotatus*) [2,13,15]. According to the same references, of these genera, *Aedes* and *Culex* are considered as the most important from the point of view of vector competence and abundance. In Europe, several potential RVFV vectors are currently present. For instance, *Aedes vexans vexans*, *Ochlerotatus caspius*, and *Culex pipiens* have a known distribution in the United Kingdom [2,23]. From a disease exposure point of view, continued vector importation events from RVF-affected countries, in combination with climatic and environmental changes, could increase the likelihood of the disease vectors being established and adapted to new environments [27]. Additionally, climatic, environmental, and genetic changes could result in changes in competence and capacity (i.e., external factors such as number and lifespan of the vector, feeding preferences of the host) of local (European) potential vectors [23], enabling them to transmit the virus once infected. Therefore, in this study we were interested in mosquitoes of any species considered as RVFV vectors that have been found aboard direct flights (see Section 4.2.4.) from the countries considered (see the Section 4.2.1) to European countries.

#### 4.2.3. Spatio-Temporal Characteristics of RVFV

The epidemiological cycle of RVFV has been mainly reported in three distinct environments (i.e., in the dambos, semi-arid, and irrigation regions). First, in the dambos regions (i.e., shallow depressions providing an ideal mosquito habitat when flooded) seen in eastern and southern Africa, the transmission cycle of RVFV depends on rainfall, and the beginning of the epidemic period is correlated with heavy precipitation, often linked with the El Nino Southern Oscillation [3,28,29]. In dry seasons, the maintenance of the virus may be linked to transovarial transmission of the *Aedes mcintoshi* mosquito [30,31].

Secondly, in semi-arid regions, found in West Africa, the mechanism for virus persistence remains unclear. In these regions, RVFV outbreaks have not been correlated with increased rainfall, and were often observed during years of rainfall deficit [32,33,34]. Previous research suggests that a rainless period lasting at least for a week, followed by large precipitation at the end of rainy season might have triggered RVF outbreaks in Mauritania and northern Senegal [35,36,37]. In dry seasons, the virus might be maintained due to transovarial transmission of *Aedes mcintoshi* as demonstrated in East Africa or through an unknown wildlife reservoir [15,29].

Thirdly, in irrigation regions, permanent bodies of water enable year-long transmission of RVFV mainly through *Culex* species mosquitoes [29]. The RVFV transmission mechanisms have also been assessed in a temperate and mountainous area of Madagascar [38,39]. Given the distinct epidemiological cycle of RVFV in different ecological systems and the availability of entomological data only from outbreaks that occurred in dambos regions in Kenya and Sudan [40,41], this model provides estimations specifically for East Africa.

#### 4.2.4. Flights

In this model, only direct flights originating from the countries considered (see the Section 4.2.1.) and flying to European countries were taken into account. Since it has been observed that mosquitoes can survive long-distance flights [42], some European countries (e.g., United Kingdom (UK), France, Italy), require disinsection of selected flights originating from African countries as a preventive measure mainly against malaria, yellow fever, and dengue fever. Given the geographic distribution of these diseases, the disinsection policy also covers flights from countries previously affected by RVF. Despite these measures, it has been shown that mosquitoes are able to survive long-distance flights in aircraft flying from Africa to European countries with a disinsection policy (e.g., [43,44]).

Only flights accomplished during the period May to October were considered. This is the period when mosquito populations, including RVFV potential vectors, are present in European countries [45]. Therefore, from the perspective of a potential disease spread, this would be the riskiest period for an epidemiological cycle of RVF to be formed in a European country, under specific circumstances (e.g., environmental changes, presence of ruminants). This timeframe also includes the timing of occurrence of most RVF outbreaks, as has been seen in East (vaguely from December to June [3,46]) and West African countries (vaguely from September to November [34,47]). Thus, this would be the riskiest period for mechanical transportations of RVF-carrying mosquitoes to European countries to occur. It should also be highlighted that the calculation of this model is not for a random year period but for a year period in which an epidemic occurs in Africa, since there are inter-epizootics periods of 5–15 years.

### 4.3. Hazard Identification

Given the aforementioned considerations, the hazard in this study was considered to be the RVFV-infected mosquitoes that are mechanically transported by planes, in direct flights from affected countries (as previously defined) to European countries, in the case of an outbreak in affected countries. These mosquitoes belong to species that are known vectors of RVFV, even if not currently present in Europe. The introduction of the virus into European countries via this pathway is an essential and fundamental prerequisite for the potential transmission of the virus within these countries and, therefore, the introduction of the disease. As previously described, the model was parametrized for the case of flights from East African countries to the United Kingdom and for the period between May and October.

### 4.4. Model Description and Equations

**Figure 3:**
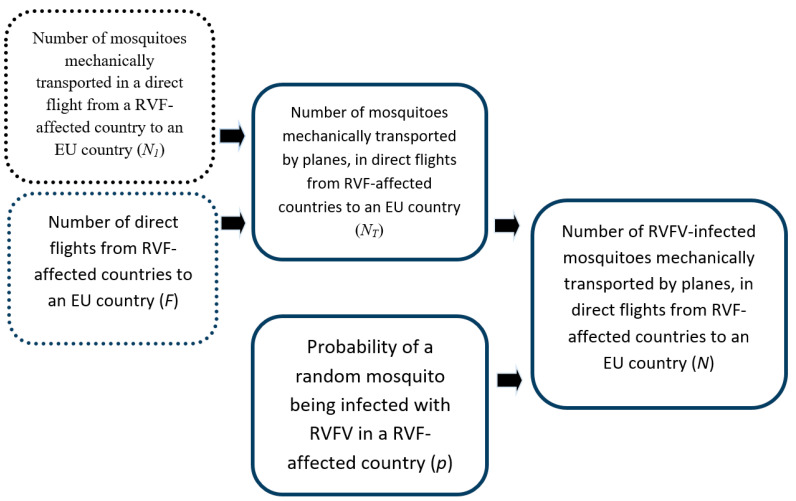
Model framework describing the introduction of RVFV-infected mosquitoes into an EU country, mechanically transported by commercial planes originating from RVF-affected countries.

The model pathway describing the introduction of RVFV into a European country via virus-infected mosquitoes on planes is presented in Figure 3. Table 2 summarizes the variables, formulas, and values used in the model.

#### 4.4.1. Probability of a Random Mosquito Being Infected with RVFV in an RVF-Affected Country (*p*)

To estimate the probability of a mosquito being infected with RVFV in an RVF-affected African country (*p*), data on the mosquito infection rates is required. The methodology of entomological studies estimating infection rates in mosquitoes can vary. Most studies on RVF surveillance have done so by grouping mosquito samples into pools (e.g., [41]), while others have used individual mosquito testing (e.g., [40]). Therefore, the probability of a random mosquito testing positive (i.e., infected with RVFV) during an entomological study undertaken in an affected country, can be estimated in different ways (see the following sections “Individual mosquito testing” and “Mosquito pool testing with constant pool size”). As described in the Section 4.2”, in this study we took into account the countries as a whole, and not just the regions where outbreaks have occurred.

##### Individual Mosquito Testing

In the case of the availability of data from individual mosquito testing (case one), the uncertainty over the probability of a random mosquito testing positive (i.e., infected with RVFV), denoted $p_{1}$ for case one, can be estimated using a Beta distribution:

(1) $$p_{1}\;\;\;\sim \;Beta\left(s+1,\;v-s+1\right),\;$$

where *s* is the number of infected mosquitoes and *v* is the total number of mosquitoes tested.

##### Mosquito Pool Testing with Constant Pool Size

In the case of the availability of data from mosquito pool testing where the pool size is constant (case two), the statistical estimation of the probability of a random mosquito testing positive (i.e., infected with RVFV), denoted $p_{2}$ for case two, can be based on testing results using the maximum likelihood estimation (MLE), as developed by [51] (expressed by [48]):

(2) $$p_{2}=MLE=1-{\left(1-\frac{Y}{X}\right)}^{1/l}$$

where *Y* is the number of positive pools, *X* is the number of pools tested, and *l* is the pool size.

##### Probability of a Random Mosquito Being Infected with RVFV in an RVF-Affected Country (p)

As described in the section “Considerations and assumptions”, in this study we took into account the countries as a whole, and not just the regions where outbreaks have occurred. Given this assumption, the uncertainty around the probability of a random mosquito being infected with RVFV in an RVF-affected country (*p*) can be estimated as follows:

*p* ~ Uniform (min, max), (3)

where min and max are from {*p*_1_, *p*_2_, …} and estimated using Equations (1) and (2).

To illustrate the description of this model, the probability of a random mosquito being infected with RVFV in RVF-affected East African countries (*p*) was calculated using Equation (3) (Table 2). For the parametrization of this model, data from previous entomological studies performed in Kenya [41] and Sudan [40] were used. During the entomological studies undertaken in Sudan, individual mosquitoes were tested, thus Equation (1) was used to estimate prevalence for this country (*p*_1_). During the studies performed in Kenya, mosquito pools of assumed constant size were tested and, therefore Equation (2) was used to estimate prevalence for this country (*p*_2_).

#### 4.4.2. Number of Mosquitoes Mechanically Transported by Planes, In Direct Flights from RVF-Affected Countries to a European Country (*N_T_*)

The number of mosquitoes mechanically transported by planes, in direct flights from RVF-affected countries to a European country (*N_T_*) can be estimated as follows:

(4) $$N_{T}=N_{1}*F\;\left(4\right)$$

where *N*_1_ is the number of mosquitoes mechanically transported in a direct flight from an RVF-affected country to a European country and *F* is the average number of flights to a European country from RVF-affected countries during a time period of interest.

In order to estimate the uncertainty around the number of mosquitoes mechanically transported in a direct flight from an RVF-affected country to a European country (*N*_1_), a Discrete distribution can be employed (Table 1).

To parametrize this model, data from three available studies [43,44,49] were used to estimate *N_1_*. In the first study, Karch et al. (2001) [43] performed a survey in Roissy airport (France) from mid-August to the end of September 2000. France is a country requiring disinsection of flights from malarian countries, that coincide with countries previously affected by RVF. For their study, 42 aircraft, all arriving from tropical Africa, were examined on arrival and in total 6 live mosquitoes were found, divided in three different flights. In the second study, Hutchinson et al. (2005) [44] searched for mosquitoes in 52 aircraft that had flown from Africa and arrived at Gatwick airport (United Kingdom (UK)). UK is also a country requiring disinsection of flights from the same countries as France. In this study 52 aircraft were searched and 3 live mosquitoes were found, in three flights searched. In the third study, Scholte et al. (2014) [49] searched 38 aircraft from overseas airports immediately after landing at Amsterdam Schiphol airport, the Netherlands (2010 and 2011). Thirteen live mosquitoes in total (with a maximum of 3 mosquitoes found per aircraft) were collected in 10 aircraft, all originating from Africa. The authors mentioned that the Royal Dutch Airlines (KLM) follows a disinsection policy to control possible disease vectors when leaving designated countries, but highlighted that the policy of other flying companies is unknown.

Moreover, given the fact that the illustration of the model is performed in the case of a country with a disinsection policy (i.e., the UK), we took into account a potentially lower probability of no mosquitoes being found than what is seen in the three studies considered for the parametrization (i.e., 0.5). However, this was decided in order to include the case that disinsection is not, or is not properly, performed.

Given our previously described considerations and assumptions, this model calculated (Table 2) and considered the average number of flights to the UK from RVF-affected countries of East Africa (assuming an equal *p* in these countries), from May to October. For this, data from the Civil Aviation Authority on the number of passengers aboard these flights were used [50] (Table 2).

### 4.5. Model Implementation

The model was implemented in @Risk 7 (© Palisade Corporation, Ithaca, NY, USA), an add-on package within Microsoft Excel 2016 (© Microsoft, Redmond, WA 98052, United States). The model was run for 50,000 iterations to allow for convergence of the mean within 3%.

The model output, that is the number of RVFV-infected mosquitoes mechanically transported by planes, in direct flights from RVF-affected countries (in this case estimated for East African countries) to a European country (in this case estimated for the UK as an example of RVF-free country) (*N*) was estimated as follows:

(5) $$N=Binomial\left(N_{T},\;p\right)$$

where $N_{T}$ is the number of mosquitoes mechanically transported by planes, in direct flights from RVF-affected countries to a European country (Equation (4)) and *p* is the probability of a random mosquito being infected with RVFV in an RVF-affected country (Equation (3)).

## 5. Conclusions

Using a stochastic approach, we estimated the risk of RVFV introduction between May and October (a period when mosquito populations, including RVFV potential vectors, are present in European countries), into previously unaffected areas (e.g., United Kingdom, UK) via virus-carrying vectors traveling in commercial aircraft from RVF-affected countries (e.g., East Africa) as low but not negligible. The model developed should be easily scaled up to other European countries by amending appropriately country-specific variables in order to map the areas/airports of higher risk and inform risk management per country accordingly in order to adopt risk-mitigation measures.

## Figures and Tables

**Table 1:** Summary of the results of the model per input (*p*, *N*_1_, *N_T_*) and output (*N*) variables (min, max, mean, and mode values). In this study, given the availability of data, the calculations were made considering flights from RVF-affected East African countries to the UK.

Variable	Description	Min	Max	Mean	Mode
*p*	Probability of a random mosquito being infected with RVFV in an RVF-affected East African country ^1^	0.02	0.09	0.06	0.07
*N_1_*	Number of mosquitoes mechanically transported in a direct flight from an RVF-affected East African country to the UK	0	3	1.1	0
*N_T_*	Number of mosquitoes mechanically transported by planes, in direct flights from RVF-affected East African countries to the UK ^2^	0	3 213	1 178	0
*N*	Number of RVFV-infected mosquitoes mechanically transported by planes, in direct flights from RVF-affected East African countries to the UK ^3^	0	337	68	0

Legend: ^1^ The values for *p* are rounded to two decimals. These estimations are made assuming that an epidemic occurs in the affected area (in the case of an inter-epidemic period, *p* would equal zero). ^2^ For the period between May and October. ^3^ These estimations are made assuming that an epidemic occurs in the affected area and is estimated for the period between May and October.

**Table 2:** Summary of the variables and formulas of the model.

Variable	Description	Formula/Value(s)	Reference
Probability of a random mosquito being infected with RVFV in an RVF-affected country (*p*)			
$p_{1}$	Probability of a random mosquito being infected with RVFV in an RVF-affected country—individual mosquito testing data	$\;\;\;\sim \;Beta\left(s+1,\;v-s+1\right)$	
s	Number of infected mosquitoes from affected area	32	[40]
v	Total number of mosquitoes tested from affected area	354	[40]
MLE	Maximum likelihood estimation of prevalence of RVFV in mosquitoes	$1-{\left(1-\frac{Y}{X}\right)}^{1/l}$	[48]
*Y*	Number of positive mosquito pools	23	[41]
*X*	Number of mosquito pools tested	105	[41]
l	Pool size	10	Assumed (based on [41])
$p_{2}$	Probability of a random mosquito being infected with RVFV in an RVF-affected country—mosquito pool testing with constant pool size data	$p_{2}=MLE\;$	
*p*	Probability of a random mosquito being infected with RVFV in an RVF-affected country	Uniform (min, max), where min and max are from {*p_1_* and *p_2_*}	
Number of RVFV-infected mosquitoes mechanically transported by planes, in direct flights from RVF-affected countries to an EU country (*N*)			
*N_1_*	Number of mosquitoes mechanically transported in a direct flight from an RVF-affected country to an EU country	~Discrete({0,1,2,3},{0.5,0.1,0.2,0.2})	[43,44,49]
*Ps_1_*	Number of passengers from RVF-affected countries, from May to October aboard direct flights to an EU country	e.g., From East Africa ^1^ to the UK: 132 750	[50]
*Ps*	Average number of passengers per flight	124	Assumed (based on data from airlines and aircraft size)
*F*	Average number of direct flights to an EU-country from RVF-affected countries, during a specified time period	$F=\frac{Ps_{1}}{Ps}$	
*N_T_*	Number of mosquitoes mechanically transported by planes, in direct flights from RVF-affected countries to an EU country	$N_{T}=N_{1}*F$	
*N*	Number of RVFV-infected mosquitoes mechanically transported by planes, in direct flights from RVF-affected countries to an EU country (N)	$N=Binomial\left(N_{T},\;p\right)$	

Legend: ^1^ Flights from Burundi, Djibouti, Ethiopia, Kenya, Rwanda, Somali Republic, Sudan, Tanzania, Uganda.

## Data Availability

The data that support the findings of this study are available from the corresponding author upon reasonable request.
